# Patient Satisfaction with Pharmacist-Led COVID-19 Testing in Community Pharmacies: Insights from the Croatian Experience

**DOI:** 10.3390/healthcare13070693

**Published:** 2025-03-21

**Authors:** Katarina Fehir Šola, Pero Hrabač, Urszula Religioni, Ljubica Frančić Pranjković, Piotr Merks

**Affiliations:** 1 Pharmacy of Bjelovar, Bjelovar, Croatia, 43000 Bjelovar, Croatia; 2 Faculty of Medicine, University of Josip Juraj Strossmayer Osijek, 31000 Osijek, Croatia; 3 “Andrija Stampar” School of Public Health, School of Medicine, University of Zagreb, 10000 Zagreb, Croatia; 4 School of Public Health, Centre of Postgraduate Medical Education of Warsaw, 01-813 Warsaw, Poland; urszula.religioni@gmail.com; 5 Pharmacy Vaše Zdravlje, 10000 Zagreb, Croatia; ljubica.francicpranjkovic@vasezdravlje.hr; 6 Faculty of Medicine, Collegium Medicum, Cardinal Stefan Wyszyński University, 01-815 Warsaw, Poland

**Keywords:** community pharmacy services, COVID-19, COVID-19 testing, pharmacists

## Abstract

**Background/Objective:** Pharmacists are among the most accessible healthcare professionals, playing a crucial role in public health. In response to the ongoing COVID-19 pandemic, many countries, including Croatia, have expanded the responsibilities of pharmacists. Since November 2021, Croatian pharmacists have been authorized to provide COVID-19 testing in community pharmacies. This study explores patients’ perceptions of these pharmacy-based testing services. **Methods:** This study employed a quantitative research approach, utilizing a structured questionnaire as the primary data collection tool. The research was conducted between November 2021 and January 2022, with a total of 211 participants from diverse backgrounds. **Results:** The findings demonstrate a highly positive perception of COVID-19 testing services in community pharmacies. A significant majority (95.2%) of patients found pharmacist-administered testing to be convenient. Additionally, 92.3% believed that pharmacists possess the necessary skills to perform the tests, and 94.7% expressed willingness to undergo testing in a pharmacy again. Notably, 94% of respondents indicated their intention to utilize pharmacists’ services for future testing. **Conclusions:** Croatian patients exhibit a strong positive attitude towards pharmacist-led COVID-19 testing in community pharmacies. These findings highlight the pivotal role of pharmacists in enhancing healthcare accessibility and demonstrate the value of integrating pharmacy-based testing services into public health strategies. This study provides valuable insights into the evolving role of pharmacists in healthcare delivery.

## 1. Introduction

In 2020, the COVID-19 pandemic was declared, prompting bold public health measures to mitigate its impact. Pharmacists are among the most accessible healthcare professionals, playing a pivotal role in healthcare systems worldwide. Community pharmacists, in particular, have long been active contributors to public health, offering essential services beyond medication dispensing. Their willingness to expand these responsibilities and strengthen healthcare systems across Europe is well documented [1,2,3,4].

The introduction of COVID-19 testing into pharmacy services marks a significant shift in the role of pharmacists, reflecting an increased level of professional accountability. Historically, pharmacies primarily focused on dispensing medications and providing basic health consultations. However, the urgent need for widespread COVID-19 testing and the accessibility of community pharmacies led many governments, including Croatian authorities, to authorize pharmacists to perform COVID-19 tests. This policy change represents a transformative moment in pharmacy practice, integrating pharmacists more deeply into the broader healthcare framework.

Patient satisfaction is a crucial metric for evaluating the quality of healthcare services, as it is closely linked to service quality and effectiveness [5]. As a key driver of healthcare improvement, patient satisfaction can vary based on several factors, including the geographic location of pharmacies and the range of services offered.

The COVID-19 pandemic underscored the indispensable role of all healthcare professionals, whose expertise and ethical commitment became critical in crisis response. Pharmacists played a unique and essential role in this landscape, collaborating with other medical professionals to optimize patient care [6]. Their contributions extended beyond traditional pharmacy duties, encompassing health promotion, patient counseling, and direct involvement in pandemic management efforts. Many countries expanded their COVID-19 testing capacity through diverse testing sites, including hospitals, clinics, pharmacies, schools, and public venues such as sports complexes and religious institutions [7,8].

The testing procedure used in pharmacies primarily involved rapid antigen tests, which detect SARS-CoV-2 viral proteins through lateral flow immunoassays. These tests demonstrated high specificity (93.9–100%) and sensitivity (84.0–97.6%) when compared to RT-PCR (Reverse Transcription Polymerase Chain Reaction), which served as the reference standard for detecting SARS-CoV-2 infection [9,10]. Rapid testing proved particularly useful for individuals exposed to confirmed COVID-19 cases and for monitoring infections in high-risk settings. Repetitive testing helped identify infected individuals early, preventing further transmission and supporting public health measures such as social distancing [8,11]. Patients who tested positive were advised to follow national public health guidelines, which included self-isolation and reporting their results to the appropriate health authorities. They were also instructed to consult a healthcare professional for further evaluation and possible confirmatory testing if required.

The pandemic introduced unprecedented challenges in controlling infectious diseases, emphasizing the need for effective collaboration among healthcare professionals. Since the outbreak, pharmacists worldwide have actively engaged in protecting public health, reinforcing their role as integral members of the healthcare system [11].

Recognizing the potential of community pharmacists in pandemic response, the Croatian government took decisive action in November 2021 during the public health emergency. Licensed pharmacists, who in Croatia are professionals who have completed pharmaceutical studies at a recognized university and passed a state professional exam, which entitles them to practice independently, were granted the authority to conduct and oversee COVID-19 testing. Initially, pharmacies were integrated into the national network responsible for issuing EU Digital COVID Certificates. This regulatory expansion allowed pharmacists—following specialized training—to provide COVID-19 testing services. The Croatian Health Insurance Fund (CHIF), in collaboration with the Croatian Chamber of Pharmacists (CCP), facilitated pharmacist education and ensured the seamless implementation of these services.

This study aimed to assess patient perceptions of COVID-19 testing services provided in community pharmacies in Croatia. By examining patient experiences, the study sought to highlight pharmacists’ ability to offer expanded healthcare services and their contribution to strengthening the public health system.

## 2. Material and Methods

This study received ethical approval from the Ethics Committee of Pharmacy Bjelovar (UR.BR.-23587/22). It was conducted between December 2021 and January 2022 at Pharmacy Bjelovar in Croatia. The research aimed to assess patient perceptions and satisfaction with COVID-19 testing services provided by pharmacists. Pharmacists who have completed pharmaceutical studies at a recognized university and passed a state professional exam, which entitles them to practice independently, have the right toprovide COVID-19 testing. After obtaining their degree, pharmacists must complete a mandatory one-year internship in a pharmacy, after which they take a state exam organized by the Ministry of Health to obtain a license. The license is issued by the Croatian Chamber of Pharmacists (*Hrvatska Ljekarnička Komora*) and is renewed every six years, during which pharmacists must participate in continuing education courses to accumulate the required number of points necessary for license renewal.

### 2.1. Study Design and Participants

A cross-sectional, questionnaire-based study was employed to collect data from individuals who underwent COVID-19 testing using rapid antigen tests, at the pharmacy. The selection of pharmacies for this study was based on accessibility and feasibility. One pharmacy located in the county capital (urban setting) and another in a nearby rural town were chosen to provide a comparative perspective on patient satisfaction in different healthcare settings. The selection was influenced by the willingness of the pharmacies to participate and their capacity to conduct pharmacist-led COVID-19 testing. This approach allowed for an initial assessment of pharmacy-based testing services while considering geographical diversity. The inclusion criteria for participation were as follows:

− Being 18 years of age or older.
− Having received a COVID-19 test performed by a pharmacist at the pharmacy.
− Providing informed consent to participate in the study.

All eligible patients who received a test during the study period were invited to participate, and only those who consented were included. Informed consent was obtained from each participant before they completed the survey, ensuring voluntary participation and adherence to ethical research standards.

### 2.2. Development and Validation of the Questionnaire

A specially designed anonymous questionnaire was developed as the primary research tool for this study. The instrument consisted of 13 multiple-choice questions, requiring participants to select a single response from the options provided. The questionnaire was adapted, with permission from the original authors, from a validated survey assessing patient satisfaction with pharmacist-led vaccination services.

To ensure the reliability and validity of the questionnaire, the research team conducted a comprehensive literature review and sought expert input. Four specialists in pharmacy research methodology and public health were consulted to refine the questionnaire. Their insights helped ensure that the questions were appropriately structured, relevant to the study objectives, and capable of accurately capturing patient perceptions and experiences.

Additionally, academic staff and practicing pharmacists reviewed and validated the initial version of the questionnaire. Their feedback led to refinements in wording, clarity, and content, ensuring that the questions were both understandable to participants and methodologically sound. The final version of the questionnaire was divided into three key sections:

− Demographic Information: Collected data on gender, year of birth, level of education, marital status, and place of residence.
− Patient Satisfaction with Testing Services: Assessed participants’ experiences, opinions, and satisfaction with COVID-19 testing conducted by pharmacists.
− Additional Questions: Included supplementary inquiries related to patient perceptions of pharmacists’ role in healthcare and their willingness to use pharmacy-based testing services in the future.

### 2.3. Data Collection Procedure

Patients who met the inclusion criteria were approached immediately after completing their COVID-19 test at the pharmacy. They were provided with detailed information about the study and invited to participate. Those who agreed to take part completed the questionnaire on-site in a designated area within the pharmacy, ensuring privacy and minimizing response bias. The survey was self-administered, allowing participants to complete it independently. However, pharmacists were available to clarify any doubts if needed.

To further encourage participation, patients were reassured that their responses would remain completely anonymous and that their personal data would not be recorded. No identifying information was collected, ensuring confidentiality.

### 2.4. Study Sample

This study analyzed a convenience sample of 211 individuals who underwent COVID-19 testing conducted by pharmacists in Bjelovar-Bilogora County, located in central Croatia. According to the 2021 census, the county spans an area of 2640 square kilometers and has a total population of 102,205. The administrative center of the county is the city of Bjelovar, which has 36,316 inhabitants.

The study participants were tested at one of two pharmacy locations:

The majority (n = 167, 79.1%) were tested at a pharmacy in central Bjelovar, representing an urban setting.

The remaining participants (n = 43, 20.9%) were tested at a pharmacy in the village of Velika Pisanica, a rural area with a population of 1313.

Table 1 provides an overview of the sample distribution by location.

### 2.5. Ethical Considerations

The study followed ethical principles outlined in the Declaration of Helsinki. Ethical approval was obtained from the relevant committee, and all participants provided informed consent before completing the survey. Participation was voluntary, and individuals had the right to withdraw at any time without consequences. Additionally, data were handled securely and used solely for research purposes.

By employing a rigorous methodological approach, this study aimed to generate robust and reliable insights into patient perceptions of pharmacist-led COVID-19 testing services in Croatia.

### 2.6. Data Analysis

The collected data were analyzed using the Jamovi statistical software package (The jamovi project, 2022, Version 2.3). Retrieved from https://www.jamovi.org (accessed on 1 February 2022).

Categorical variables (nominal or ordinal data) were analyzed descriptively using contingency tables to summarize distributions. To assess statistical significance, the chi-square test was applied, with Yates’ correction used where appropriate to adjust for small sample sizes and improve accuracy.

Continuous variables (measured on a ratio scale) were first tested for normality using the Shapiro–Wilk test. Based on the results, if data followed a normal distribution, parametric tests such as the t-test (for two-group comparisons) or ANOVA (for multiple-group comparisons) were applied; if data deviated from normality, non-parametric tests such as the Mann–Whitney U test (for two independent groups) or the Kruskal–Wallis test (for multiple groups) were used.

All statistical tests were two-tailed, and results were interpreted with a significance level (α) set at 0.05. A *p*-value < 0.05 was considered statistically significant, indicating that the observed differences were unlikely to have occurred by chance.

This structured analytical approach ensured a rigorous evaluation of the data, allowing for meaningful interpretations of patient perceptions regarding pharmacist-led COVID-19 testing in community pharmacies.

## 3. Results

Participants’ experiences with COVID-19 testing provided by pharmacists were assessed by examining their perceptions of the service’s convenience, the competence of pharmacists in conducting the tests, and their willingness to use similar pharmacy-based services in the future.

The responses, presented in Table 2, indicate a generally high level of satisfaction with the service.

In addition to evaluating patient satisfaction, the study also explored participants’ recent health history. This included an assessment of whether they had experienced symptoms indicative of COVID-19 in the past six months, any instances of sick leave taken due to such symptoms, and any adverse reactions they may have encountered following COVID-19 vaccination.

The responses revealed that the majority (n = 189, 90.0%) did not report experiencing any symptoms suggestive of COVID-19. Among those who had symptoms, the most frequently reported included flu-like symptoms (n = 7, 3.3%), loss of smell and taste (n = 4, 1.9%), and high fever (n = 1, 0.5%). Nine participants (4.3%) reported experiencing all listed symptoms.

Similarly, 190 participants (90.5%) reported that they had not been on sick leave in the past six months. Among the 20 participants (9.5%) who had taken sick leave, the majority (n = 16, 7.6%) were absent from work for up to 15 days.

Regarding adverse reactions to COVID-19 vaccination, 17 participants (8.1%) reported experiencing post-vaccination side effects. The most commonly reported adverse effect was muscle pain (n = 11, 5.2%).

Table 3 provides a detailed breakdown of responses to these health-related questions.

Although participants generally expressed high levels of agreement with all patient satisfaction statements in Table 2, further analysis was conducted to determine whether responses varied based on demographic factors, including location, gender, age, and education level.

Comparison of responses between urban and rural participants revealed that those tested in urban settings reported slightly lower levels of agreement across all patient satisfaction statements. However, these differences were not statistically significant (*p* > 0.05 for all comparisons).

Similarly, no statistically significant differences were observed in responses based on gender (*p* > 0.05 for all comparisons), although male participants tended to report lower levels of agreement than female participants.

With respect to age, older participants were generally more likely to express agreement with study statements. However, only one question—”In my opinion, the pharmacist had the skills to perform the test”—showed a statistically significant correlation with age (r = 0.120, *p* = 0.036), although the correlation was weak.

Participants with higher levels of education demonstrated a trend toward greater agreement with study statements. However, none of these correlations reached statistical significance.

Finally, no significant associations were observed between responses to the health history questions and levels of agreement with the satisfaction statements. Participants who reported experiencing COVID-19 symptoms, taking sick leave, or having adverse reactions to vaccination did not exhibit a clear tendency toward higher or lower satisfaction with pharmacy-based COVID-19 testing.

These findings suggest that patient satisfaction with COVID-19 testing who were tested in pharmacies was generally high and did not vary significantly based on demographic factors or prior health experiences.

## 4. Discussion

This study examined the patients’ satisfaction and the role of pharmacists in providing COVID-19 testing services in both urban and rural settings. The participants included individuals tested at a pharmacy in the center of Bjelovar (classified as an “urban” setting, *N =* 167; 79.1%) and those tested at a pharmacy in the nearby village of Velika Pisanica (population: 1,313; classified as a “rural” setting, *N =* 43; 20.9%).

Our findings indicate a high level of patient satisfaction with pharmacist-administered testing. Over 95.2% of respondents agreed that having pharmacists perform the test was convenient, highlighting the accessibility of pharmacy-based testing services. Additionally, 92.3% of participants expressed confidence in pharmacists’ ability to perform the test competently. These results are particularly noteworthy given that all pharmacists involved in the study had undergone training in clinical testing procedures. Furthermore, 94.7% of patients believed that pharmacists possess the necessary skills and knowledge to offer additional diagnostic tests in the future.

Despite this strong acceptance and trust in pharmacists’ clinical capabilities, their role in healthcare remains underutilized. In many countries, pharmacists are integrated into multidisciplinary healthcare teams, actively contributing to patient care beyond medication dispensing. Expanding their responsibilities to include diagnostic testing within pharmacies could enhance healthcare accessibility and efficiency. Pharmacist-led testing offers timely results and may alleviate pressure on other healthcare providers by streamlining diagnostic services within the community [12]. SARS CoV-2 is a highly contagious virus, and management of COVID-19 infections requires a multidisciplinary approach. Pharmacists contribute to fighting the pandemic worldwide and in Croatia. Our study aimed to assess the level of satisfaction of patients with pharmacists testing in pharmacy. The study was conducted in two pharmacies (urban and rural areas) of Pharmacy Bjelovar. We report on a convenient sample of 210 subjects who were tested for COVID-19 by pharmacists of the Bjelovar-Bilogora county in central Croatia. On 11 March 2020 the WHO declared COVID-19 as a pandemic and asked for enhanced cooperation and collaboration among different nations. On February 2020 the first confirmed case due to the novel SARS CoV-2 virus was reported in Croatia. Expanded roles for pharmacy professionals and pharmacies during the pandemic are also being outlined.

One measure of the effectiveness of pharmacy service is patient satisfaction. Numerous studies have looked into how satisfied patients are with community pharmacy services and how they feel about them [13,14,15,16,17]. Patient satisfaction is an important aspect of healthcare services. Community pharmacists are increasingly acting as care extenders to address primary care provider shortages and medication misuse, providing a range of patient care services such as emergency medications, prescription renewals, dose or formulation changes, therapeutic substitution, minor prescribing ailments, initiating treatment, prescription drug therapy, ordering and interpreting laboratory tests, and administering injectable medications [18].

To the best of our knowledge, only a few countries (regions) have introduced programs or pilot programs for COVID-19 testing by pharmacists. They include, e.g., New York, which has implemented a COVID-19 testing pilot program that includes independent pharmacies. The project was implemented in cooperation with the Pharmacists Society of the State of New York and the state’s network of Community Pharmacy Enhanced Services Network [19]. Although the government program has been running since April 2020, there is not a comprehensive listing of US pharmacy testing. However, it is estimated that if COVID-19 tests were available in all US pharmacies, an estimated 94% of the US population would be willing to use them [5].

Canada is the second region to introduce COVID-19 testing. Research conducted here shows that 99% of patients are satisfied with the tests conducted [20]. Pharmacists are a highly educated group of medical professionals, providing a wide range of services in the field of pharmaceutical care, including consultancy, also in the field of vaccinations, which is emphasized by numerous studies. The availability of pharmacies, their opening hours and the distance from the places of residence/work of many patients should be additionally emphasized, which is also a great advantage in terms of the possibility of reaching these facilities. The trust that patients have towards pharmacists is also important, which is also emphasized in many studies [21,22,23,24,25,26]. All these elements may indicate the important role of public pharmacies in providing a wide range of health services in order to protect public health.

Even though the COVID-19 pandemic has ended, our study is still relevant. It shows the role of pharmacists in the health care system and the possibility of providing additional services to patients. Our respondents showed high patient satisfaction with the services provided by pharmacists, as well as willingness to use other services in pharmacies. These results indicate the important role of community pharmacies and pharmacists, who in the face of health threats can play an important role in protecting patients’ health.

## 5. Limitations of the Study

Our study has several limitations. First of all, we had no influence on the distribution of demographic characteristics among the study participants. Therefore, the obtained results and findings cannot be generalized to the entire population of the country. The current study design was the only way to achieve the stated purpose of the study. However, alternative designs can be used to obtain a more nationally representative sample.

Another limitation of this study is the lack of existing research on pharmacy testing services. The paucity of previous research in this area highlights the early stage of development of this field. As a result, the study may encounter difficulties due to limited comparative data and a less established research framework. Therefore, there is a strong need for further large-scale research to thoroughly investigate and validate the role of pharmacies in the provision of testing services.

Additionally, calculating the minimum required sample size was challenging due to the novelty of the topic and the limited availability of prior studies on pharmacist-led COVID-19 testing. As a result, we based our approach on practical feasibility rather than strict statistical power calculations. We acknowledge this as a methodological limitation and emphasize the need for future research with larger, more systematically determined sample sizes to enhance the generalizability of findings.

## 6. Conclusions

This study confirms that patients who are tested in pharmacy are highly satisfied with pharmacist-led COVID-19 testing and are open to utilizing similar pharmacy-based services in the future. Expanding pharmacists’ roles to include diagnostic testing and preventive care, such as vaccinations, can enhance healthcare accessibility and efficiency.

Pharmacists played a key role during the COVID-19 pandemic, and their continued integration into public health initiatives is essential. High patient satisfaction with pharmacy-led services highlights the potential for pharmacists to contribute further to disease prevention and healthcare delivery. Strengthening their involvement in interdisciplinary teams can improve patient outcomes and public health protection.

## Figures and Tables

**Table 1:** Basic demographic properties of the subjects.

Parameter	N (%)
**Gender**	
M	100 (47.4%)
F	111 (52.6%)
**Education**	
Elementary school	8 (3.8%)
High school	137 (65.2%)
College	33 (15.7%)
University and above	32 (15.2%)
**Marriage status**	
Married/partnership	120 (57.4%)
Unmarried	73 (34.9%)
Divorced	14 (6.7%)
Widowed	2 (1.0%)
**Setting**	
Urban	167 (79.1%)
Rural	43 (20.9%)

**Table 2:** Proportions of the answers to each statement the participants were asked.

**It is convenient for your pharmacist to perform the test**		
**Category**	**Counts**	**% of Total**
Somewhat disagree (2)	7	3.3%
Neither agreed nor disagreed (3)	1	0.5%
Somewhat agree (4)	2	1.0%
Strongly agree (5)	200	95.2%
**In my opinion, the pharmacist had the skills to perform the test**		
Somewhat disagree (2)	12	5.7%
Neither agreed nor disagreed (3)	1	0.5%
Somewhat agree (4)	3	1.4%
Strongly agree (5)	193	92.3%
**If possible, I will also do the following COVID-19 test at the pharmacy**		
Somewhat disagree (2)	7	3.3%
Somewhat agree (4)	4	1.9%
Strongly agree (5)	198	94.7%
**I want pharmacists to perform other diagnostic TESTSs in the future**		
We strongly disagree with (1)	4	1.9%
Somewhat disagree (2)	18	8.6%
Neither agreed nor disagreed (3)	1	0.5%
Somewhat agree (4)	3	1.4%
Strongly agree (5)	184	87.6%

**Table 3:** Symptoms suggestive of COVID-19.

Symptoms Suggestive of COVID-19 in the Last Six Months	N	%
No	189	90.0%
Yes—All symptoms	9	4.3%
Yes—Flu-like symptoms	7	3.3%
Yes—Loss of smell/taste	4	1.9%
Yes—High fever	1	0.5%
**Have you been on sick leave because of the mentioned symptoms in the last six months**	**N**	**%**
No	190	90.5%
Yes—up to 7 days	9	4.3%
Yes—8 to 15 days	7	3.3%
Yes—16 to 30 days	2	1.0%
Yes—more than 60 days	2	1.0%
**Have you had an adverse reaction after vaccination?**	**N**	**%**
No	193	91.9%
Yes—muscle and arthritic pains	11	5.2%
Yes—other	4	1.9%
Yes—nausea	1	0.5%
Yes—fever	1	0.5%

## Data Availability

The original contributions presented in this study are included in the article. Further inquiries can be directed to the corresponding author.

## Abbreviations

CCP	the Croatian Chamber of Pharmacists
CHIF	Croatian Health Insurance Fund
